# Paradoxical Pyogenic Granuloma Associated With Ramucirumab but Not Bevacizumab: A Case Suggesting Differential Effects of VEGF Receptor and Ligand Inhibition

**DOI:** 10.1002/rcr2.70606

**Published:** 2026-05-05

**Authors:** Akina Nigi, Keisuke Iwamoto, Hidetoshi Itani, Shigeto Kondou

**Affiliations:** ^1^ Department of Respiratory Medicine Japanese Red Cross Ise Hospital Ise Japan

**Keywords:** bevacizumab, lung adenocarcinoma, pyogenic granuloma, ramucirumab, VEGF

## Abstract

Pyogenic granuloma is a benign vascular lesion that may bleed repeatedly and impair daily activities. Although ramucirumab is an anti‐angiogenic agent, paradoxical pyogenic granuloma has rarely been reported during its use. We describe a 75‐year‐old man with advanced lung adenocarcinoma who developed multiple bleeding pyogenic granulomas during docetaxel plus ramucirumab therapy. Histopathology confirmed lobular capillary proliferation consistent with pyogenic granuloma. Because the lesions repeatedly bled and reduced quality of life, they were excised and ramucirumab was discontinued. The patient was subsequently treated with docetaxel plus bevacizumab, and no recurrence or worsening of pyogenic granuloma was observed. This intra‐patient contrast suggests that VEGFR‐2 blockade and VEGF ligand inhibition may have different effects on paradoxical vascular proliferative lesions. Clinicians should recognize this rare toxicity and consider switching anti‐angiogenic strategy in selected cases.

## Graphical abstract

We report a case of multifocal pyogenic granuloma that developed during ramucirumab therapy but did not recur after switching to bevacizumab.

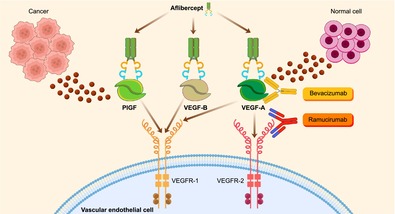

## 1 Introduction

Pyogenic granuloma (PG) is a benign vascular proliferative lesion characterized by rapid growth, friability and recurrent bleeding. Although anti‐angiogenic agents are expected to suppress angiogenesis, paradoxical PG has been reported during ramucirumab therapy [1, 2]. Ramucirumab targets vascular endothelial growth factor receptor 2 (VEGFR‐2), whereas bevacizumab targets vascular endothelial growth factor A (VEGF‐A), so these drugs inhibit the vascular endothelial growth factor (VEGF) pathway at different levels. Whether this mechanistic difference influences paradoxical vascular lesions remains unclear. We report a case of multifocal PG that developed during ramucirumab therapy but did not recur after switching to bevacizumab.

## 2 Case Report

A 75‐year‐old man was diagnosed with stage IVA lung adenocarcinoma with malignant pleural effusion in July 2022. He initially received osimertinib for EGFR exon 19 deletion–positive disease, followed by several subsequent systemic treatments because of disease progression. After multiple lines of therapy, including platinum‐based chemotherapy and immune checkpoint inhibitor–containing regimens, disease control became increasingly difficult. Before the events described below, he had received a total of 16 doses of bevacizumab.

**FIGURE 1:**
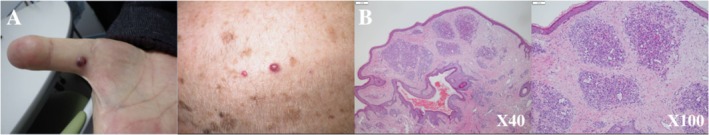
Clinical and pathological findings of pyogenic granuloma. (A) Multiple rapidly growing, friable cutaneous nodules that developed during ramucirumab therapy and were associated with recurrent bleeding. (B) Histopathological findings of a biopsied lesion showed lobulated structure, and blood vessels separated by collagen fibres are visible.

In February 2024, docetaxel plus ramucirumab was initiated as later‐line therapy for progressive pleural dissemination. After three cycles, the patient developed multiple rapidly enlarging, friable nodular lesions on the right shoulder, abdomen, retroauricular area and right thigh, accompanied by recurrent bleeding (Figure 1A). The lesions tended to worsen after each administration and partially improved during the treatment interval. After four cycles, surgical resection was performed in November 2024 because of persistent bleeding, progression of the PG and interference with daily activities, and ramucirumab was discontinued. Histopathological examination of a biopsied lesion showed lobular capillary proliferation consistent with PG (Figure 1B).

Based on the clinical course and the temporal relationship with ramucirumab administration, the lesions were diagnosed as ramucirumab‐associated paradoxical PG.

Subsequently, docetaxel plus bevacizumab was started instead of ramucirumab to maintain control of the malignant pleural effusion. During bevacizumab treatment, no recurrence or worsening of PG was observed despite continued antiangiogenic therapy. On clinical follow‐up, no new vascular proliferative lesions developed during the observation period.

## 3 Discussion

**FIGURE 2:**
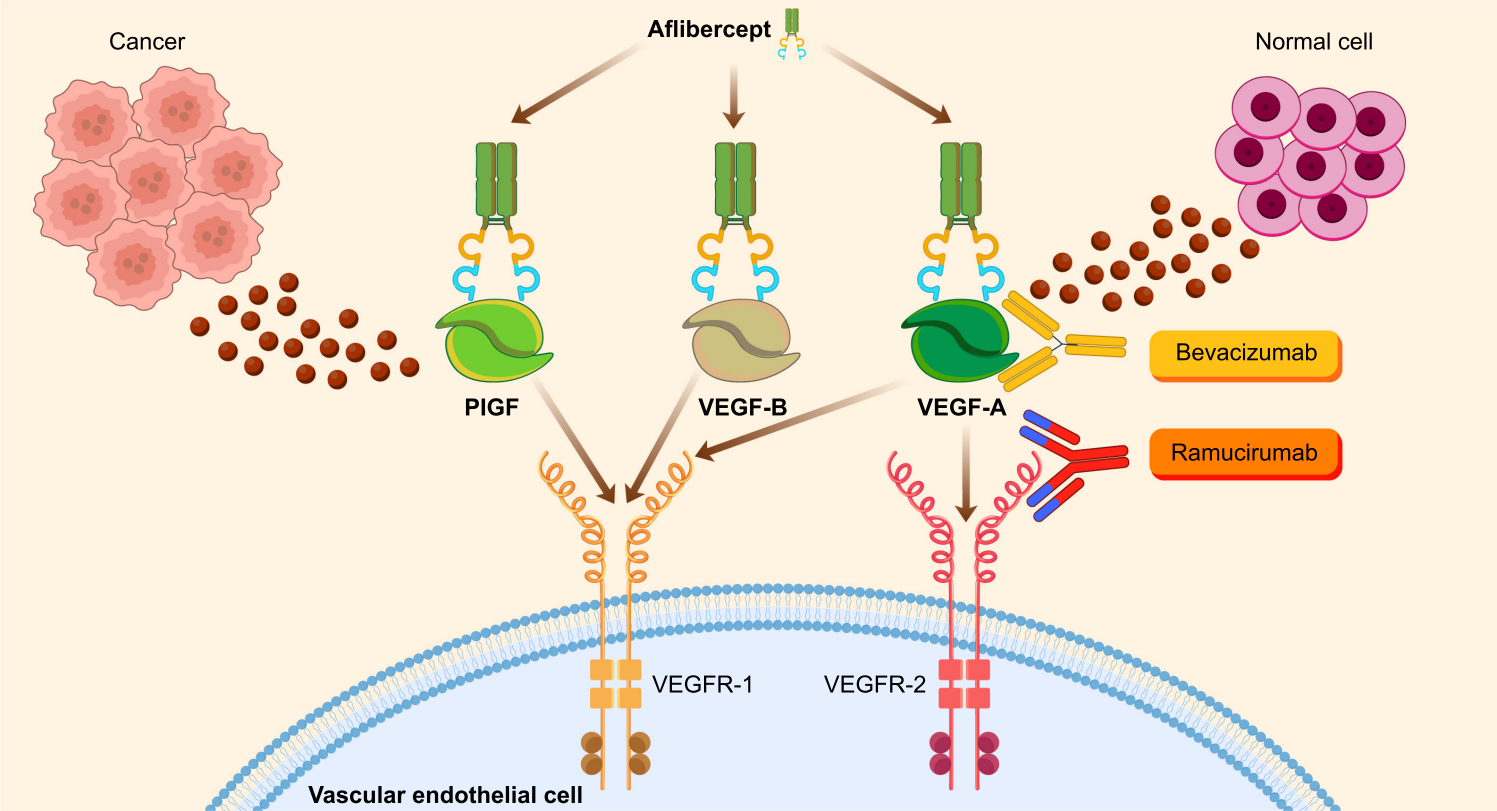
Schematic illustration of differences in target profiles among anti‐angiogenic agents. Ramucirumab blocks VEGFR‐2 at the receptor level, whereas bevacizumab neutralizes VEGF‐A at the ligand level. Aflibercept acts as a soluble decoy receptor that binds VEGF‐A, VEGF‐B and placental growth factor (PlGF).

PG is a benign but clinically significant vascular proliferative lesion that is frequently associated with bleeding and impairment of daily activities. Histopathological and immunohistochemical studies have consistently demonstrated overexpression of VEGF and increased angiogenic activity within PG lesions, supporting the central role of VEGF‐driven angiogenesis in its pathogenesis. Recently, several case reports have described the development of PG during treatment with ramucirumab [1, 2], a monoclonal antibody targeting VEGFR‐2, and this phenomenon has been described as paradoxical given the expected anti‐angiogenic effects of VEGF pathway inhibition. Figure 2 illustrates the mechanisms of VEGF receptor antibodies and VEGF antibodies [3].

In contrast, the occurrence of PG during systemic administration of VEGF ligand–targeting agents such as bevacizumab has not been clearly documented. Although PG has been reported following local or intravitreal administration of anti‐VEGF agents [4, 5], evidence of PG development or exacerbation during systemic bevacizumab therapy remains scarce. To our knowledge, direct clinical comparisons between VEGF receptor blockade and VEGF ligand neutralization within the same patient have not been previously reported.

The present case provides a unique clinical observation in which PG developed exclusively during ramucirumab therapy but did not recur or worsen during preceding and subsequent bevacizumab administration. This intra‐patient contrast suggests that the mechanism underlying PG development may differ depending on whether the VEGF pathway is inhibited at the ligand or receptor level. While VEGF ligand neutralization results in a global attenuation of angiogenic signalling, direct VEGFR‐2 blockade may lead to dysregulated or compensatory activation of alternative angiogenic pathways, resulting in localized and paradoxical vascular proliferation. Such receptor‐level imbalance rather than simple suppression of angiogenesis may contribute to the development of PG.

Although causality cannot be established from a single case, this observation supports the hypothesis that anti‐angiogenic agents targeting VEGF receptors may be more likely to induce paradoxical vascular proliferative lesions such as PG than agents that directly neutralize VEGF ligands. From a clinical perspective, this distinction may have important implications. PG associated with ramucirumab often leads to treatment discontinuation despite the therapeutic benefit of anti‐angiogenic therapy. Our case suggests that switching from receptor‐targeting to ligand‐targeting anti‐VEGF therapy may be a feasible strategy in selected patients who develop difficult‐to‐control PG. Although no direct head‐to‐head comparison exists and the anticancer efficacy of bevacizumab and ramucirumab cannot be clearly determined, the clinical benefits observed with both agents in NSCLC appear broadly comparable in their respective treatment settings. From a mechanistic perspective, switching from VEGF receptor–targeting to VEGF ligand–targeting anti‐angiogenic therapy may therefore be a biologically plausible option in selected patients who develop paradoxical vascular proliferative lesions such as PG.

This report has several limitations. It represents a single clinical observation, and mechanistic conclusions cannot be definitively drawn. Furthermore, other contributing factors such as local inflammation, microtrauma, or cumulative treatment effects cannot be completely excluded. Nevertheless, the clear temporal association and intra‐patient comparison observed in this case provide a biologically plausible and clinically relevant hypothesis that warrants further investigation. Although synergistic effects with concomitant agents cannot be completely excluded, the temporal relationship with ramucirumab administration and the absence of PG recurrence during subsequent bevacizumab treatment suggest that ramucirumab was the most likely trigger.

## Author Contributions

A.N. contributed significantly to the clinical management of the patients, study conception and manuscript writing. K.I., H.I. and S.K. are also involved in clinical practice. H.I. and S.K. contributed to pathological examination and case review. All authors reviewed the final draft of the manuscript and approved its submission.

## Funding

The authors have nothing to report.

## Ethics Statement

This study was conducted in accordance with the ethical standards of the institution. Written informed consent was obtained from the patient for participation and publication of this case report. Ethics committee approval was not required for this single‐patient case report.

## Consent

The authors declare that written informed consent was obtained for the publication of this manuscript and accompanying images and attest that the form used to obtain consent from the patient complies with the Journal requirements as outlined in the author guidelines.

## Conflicts of Interest

The authors declare no conflicts of interest.

## Data Availability

The data that support the findings of this study are available on request from the corresponding author. The data are not publicly available due to privacy or ethical restrictions.
